# Reflection- and Curriculum-Based Instruction Tuning for Diabetes-Specialized Large Language Models: Model Development and Evaluation Study

**DOI:** 10.2196/92843

**Published:** 2026-08-25

**Authors:** Jaesung Hwang, Deniise Liz Namayanja, Donghyeon Park

**Affiliations:** 1 Department of Software, Sejong University, Seoul, Republic of Korea; 2 Department of Artificial Intelligence & Data Science, Sejong University, Daeyang AI Center 707, 209 Neungdong-ro, Gwangin-gu, Seoul, 05006, Republic of Korea, 82 10-4860-7491

**Keywords:** diabetes mellitus, large language models, instruction tuning, instruction-following difficulty, curriculum learning, dietary recommendation, glycemic simulation, AI in health care

## Abstract

**Background:**

Effective diabetes management requires continuous interpretation of glycemic trends, personalized dietary guidance, and sustained patient education. Although large language models (LLMs) are increasingly being explored for health-related applications, existing general-purpose and biomedical models often struggle with diabetes-specific reasoning and instruction-following, limiting their reliability for domain-focused tasks such as clinical question answering and dietary recommendation tasks.

**Objective:**

This study aimed to develop and evaluate a diabetes-specialized LLM optimized for diabetes-specific reasoning, instruction-following, and dietary recommendation tasks.

**Methods:**

This was a model development and benchmark evaluation study. We developed a model-centric instruction refinement framework using instruction-following difficulty and reversed instruction-following difficulty to identify and replace suboptimal instruction-response pairs during instruction tuning. Curriculum-based instruction tuning was applied by sequencing instructions from lower to higher difficulty. The resulting diabetes-specialized model was evaluated across diabetes-related question answering, natural language inference, information extraction, summarization, clinical answer generation, and dietary recommendation tasks. Performance was compared with biomedical LLMs and general-purpose baselines using diabetes-specific benchmark subsets and simulation-based glycemic evaluation.

**Results:**

Across diabetes-related benchmark tasks, the proposed model demonstrated improved performance in question answering, information extraction, and generative tasks. Ablation experiments showed that the full reflection- and curriculum-based instruction tuning strategy improved overall performance by 59.03% relative to the zero-shot LLaMA3.1 8B baseline. For dietary recommendation tasks, the proposed model achieved a 0.66% improvement in the Diet Quality Index—International score compared with ChatGPT (GPT-4). Simulation-based evaluation using the SimGlucose simulator further showed that meal plans generated by the proposed model resulted in a reduced postprandial glycemic burden, as measured by a lower incremental area under the curve, compared with GPT-4−generated meal plans.

**Conclusions:**

This study demonstrates that domain-specific instruction tuning can effectively adapt general-purpose LLMs for diabetes management. By combining reflection-based instruction replacement with curriculum-based instruction tuning, the proposed approach enhances instruction-following, reasoning capability, and dietary guidance for diabetes. The results highlight the potential of specialized LLMs to provide more reliable and clinically aligned support for diabetes-related decision-making and self-management, offering a promising direction for safe and effective AI deployment in chronic disease care.

## Introduction

### Background

Diabetes mellitus remains one of the most significant global health challenges, with prevalence rates nearly doubling over the past 3 decades [1]. Managing diabetes effectively requires not only pharmacological treatment but also ongoing interpretation of blood glucose fluctuations, dietary adaptations, personalized lifestyle interventions, and continuous patient education. As the demand for scalable, patient-centered care rises, the health care community increasingly seeks robust AI tools, such as large language models (LLMs), that can support clinical decision-making and self-management.

LLMs have gained significant traction in the biomedical domain due to demonstrated advances in natural language understanding [2], large-scale incorporation of biomedical literature [3], and emerging capabilities in complex clinical reasoning tasks [4]. In the context of diabetes management, LLMs have been leveraged across a diverse set of tasks tailored to patient needs. For instance, integrated systems, such as DeepDR-LLM, combine image-based data with LLM-generated responses [5]. Retrieval-augmented generation architectures have been used to support diabetes education by grounding LLM outputs in trusted reference sources, thereby improving factual reliability [6]. Other efforts have used LLMs to analyze continuous glucose monitoring (CGM) data directly and summarize trends over time for patients and clinicians [7].

Despite these advances, existing LLM-based approaches for diabetes management still show gaps in consistency, clinical alignment, and personalization. General biomedical language models such as BioBERT (biomedical bidirectional encoder representations from transformers) [2] and BioGPT (biomedical generative pretrained transformer) [3] achieve strong performance on broad biomedical benchmarks, but they are not explicitly optimized for diabetes-specific reasoning or patient-facing counseling. Similarly, evaluations of general-purpose chatbots such as ChatGPT have identified deficiencies in diabetes-related responses, including incomplete reasoning and insufficient dietary sensitivity, thereby raising concerns about their reliability for unsupervised diabetes management support [8,9]. Collectively, these shortcomings underscore a critical gap: the absence of LLMs explicitly optimized for diabetes-specific reasoning, instruction-following, and patient-centered guidance, including accurate responses to diabetes-related queries and diabetes-appropriate dietary recommendations.

To address this gap, we propose a diabetes-specialized LLM trained on a curated, diabetes-specific instruction dataset designed to capture the clinical and practical nuances of diabetes management. Importantly, our approach moves beyond simple fine-tuning on domain-specific text. Instead, we adopt a model-centric perspective that treats the LLM as a student whose learning must be aligned with its capacity and receptiveness. Inspired by the notion of student-guided data selection introduced in selective reflection-tuning [10], we adopt a reflection-based replacement strategy using instruction-following difficulty (IFD) and reversed IFD (r-IFD) metrics to systematically filter and refine instruction-response pairs, ensuring that the training data are both cognitively challenging and instructionally coherent.

In addition, we use a curriculum-based instruction tuning framework [11] to promote stable knowledge acquisition and reduce catastrophic forgetting. By ordering training samples from lower- to higher-difficulty instructions, the model progressively acquires diabetes-specific reasoning skills while preserving foundational biomedical knowledge.

We evaluate our model across a diverse range of diabetes-specific tasks, including question answering (QA), natural language inference (NLI), and personalized dietary recommendation. Across these evaluations, the model consistently outperforms strong biomedical and general-purpose baselines. Notably, in dietary recommendation tasks measured by the Diet Quality Index–International (DQI-I) score [12], our model achieves a 0.66% improvement over ChatGPT (GPT-4), indicating superior nutritional alignment in generated meal plans.

The primary contributions of our work are as follows:

1. We adapt and apply a reflection-based filtering strategy (IFD or r-IFD) to selectively refine a diabetes-specific instruction dataset.
2. We integrate a task complexity–aware curriculum-based instruction tuning strategy that sequences diabetes-related instructions from simpler to more challenging cases, improving training stability and domain adaptation.
3. This work demonstrates strong performance across multiple diabetes-relevant tasks, including QA, NLI, IE, and dietary recommendation, and is supported by ablation experiments that evaluate the contributions of instruction optimization (IFD or r-IFD) and curriculum-based instruction tuning.
4. Finally, we assess the physiological relevance of the model’s dietary recommendations using the SimGlucose simulator [13], which uses the UVA/Padova physiological model [14] to predict glucose kinetics. Our results show reduced postprandial glycemic responses compared with those from GPT-4-generated meal plans.

Overall, this study shows that domain-aware instruction tuning, when combined with reflection-guided data refinement and curriculum learning, can effectively adapt general-purpose LLMs into clinically meaningful assistants for diabetes management, narrowing the gap between general linguistic capability and specialized clinical reasoning.

### Related Work

#### LLMs in Biomedical and Clinical Natural Language Processing

LLMs have demonstrated strong performance across a wide range of biomedical natural language processing (NLP) tasks, including medical QA, clinical summarization, and IE. Domain-adapted models such as BioBERT [2] and BioGPT [3] have shown improved performance on biomedical benchmarks by incorporating large-scale biomedical corpora during pretraining. More recent instruction-tuned biomedical models, including MedAlpaca [15], PMC-LLaMA [16], ChatDoctor [17], and BioMistral [18], have further expanded the scope of clinical language understanding by aligning model behavior with natural language instructions.

Despite these advances, most biomedical LLMs are trained to optimize general medical competence rather than task consistency within a specific disease domain. As a result, their performance often varies substantially across task types, particularly when moving from factual QA to generative or reasoning-intensive tasks. This limitation is especially pronounced in chronic disease management, where models must integrate clinical context, lifestyle constraints, and patient-specific considerations.

#### Instruction Tuning

Instruction tuning [19,20] has emerged as a widely adopted posttraining paradigm for enhancing the ability of LLMs to follow natural language instructions across a wide range of downstream tasks. Early research efforts in this area relied primarily on manually creating task-specific instruction tuning datasets [21,22]. Subsequent work has shifted toward scalable data construction strategies that leverage more powerful LLMs to generate synthetic data [23-27]. These studies have laid the foundation for instruction tuning, demonstrating that large task-specific or multitask instruction tuning datasets can substantially boost the instruction-following behavior and generalization in language models.

#### LLM-Based Approaches for Diabetes Management

Recent studies have explored the use of LLMs in diabetes-related applications, primarily focusing on patient education, lifestyle, and self-management [28-30]. Prior work has examined conversational agents that provide nutritional guidance, carbohydrate-related explanations, and guideline-informed recommendations to assist daily decision-making in diabetes care [31,32].

Other efforts have focused on using LLMs to translate CGM data into natural language summaries that improve interpretability for patients and clinicians [7,33].

Despite these advances, existing LLM-based systems for diabetes management are typically evaluated in narrow task settings or rely on general-purpose models without explicit optimization for diabetes-specific instruction-following and reasoning. To date, there has been limited work on systematically adapting LLMs to perform consistently across a diverse set of diabetes-relevant tasks, such as clinical QA, reasoning over patient scenarios, and generating diabetes-appropriate dietary recommendations, within a unified, disease-specialized framework.

## Methods

### Study Design

**Figure 1.**
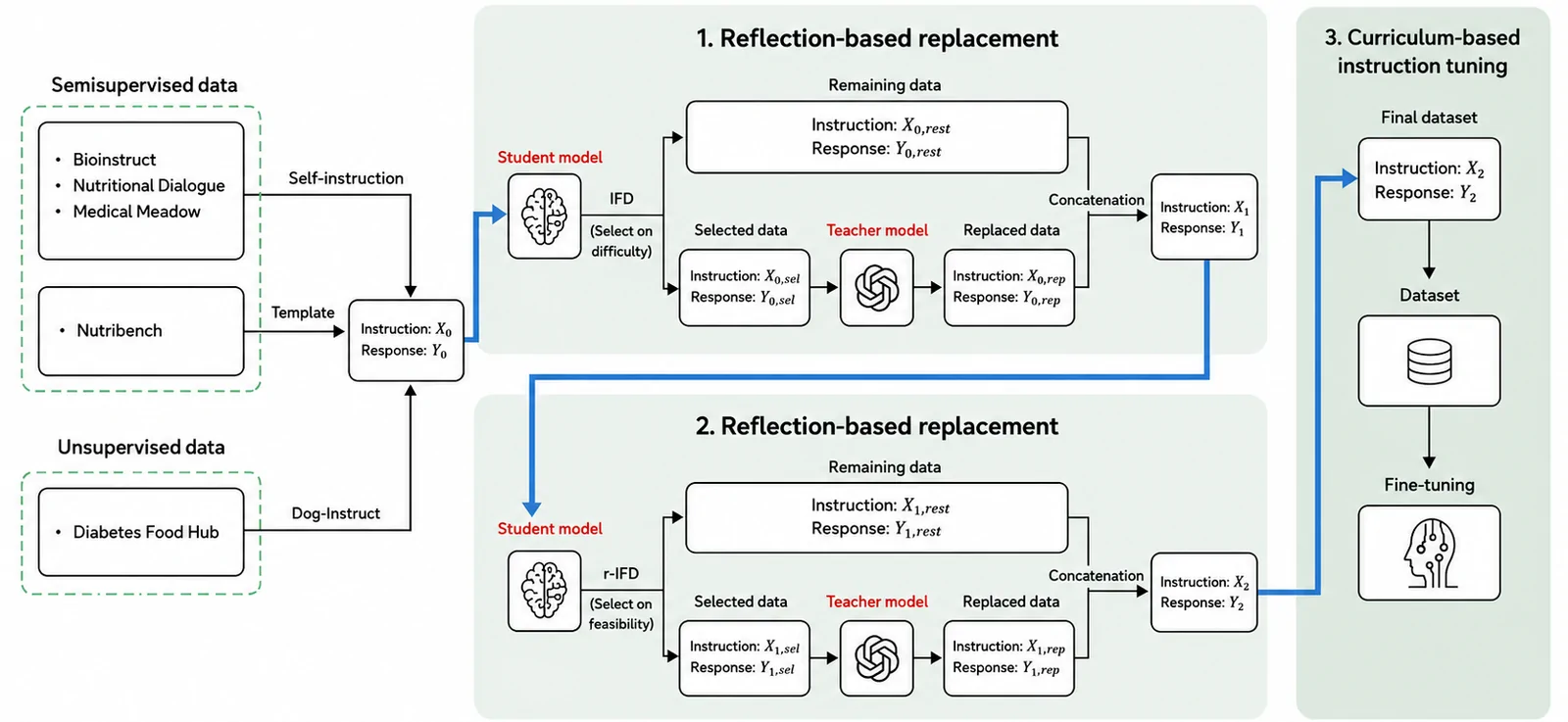
Overall architecture of the proposed diabetes-specialized large language model training pipeline, including instruction dataset construction, reflection-based replacement, and curriculum-based instruction tuning. IFD: instruction-following difficulty; r-IFD: reversed instruction-following difficulty.

To address the limitations of general biomedical LLMs in performing diabetes-specific reasoning, we developed and evaluated a diabetes-specialized LLM. The model was fine-tuned using reflection-based replacement and curriculum-based instruction tuning. Performance was assessed across multiple diabetes-related tasks, including QA, NLI, IE, summarization, generation, and dietary recommendation. An overview of the proposed instruction refinement and curriculum-based instruction tuning pipeline is shown in Figure 1.

### Instruction Dataset Construction

To construct a domain-specific instruction dataset that captures the clinical and practical complexity of diabetes management, we aggregated content from 4 complementary sources. These included BioInstruct [34], a biomedical NLP dataset comprising 25,005 instruction-response pairs generated using GPT-4; Medical Meadow [15], a collection of clinical NLP prompts covering disease definitions, treatment recommendations, and patient counseling scenarios; Diabetes Food Hub [35], a curated repository of diabetes-friendly recipes and nutritional information; and Nutribench [36], a structured nutrition dataset providing recipe-level guidance for glycemic control.

Each dataset entry was structured as a triplet consisting of (1) a natural language instruction describing the task, (2) an input argument that instantiated the instruction, and (3) an output reflecting the correct execution of the instruction. To generate new samples, we adopted the Self-Instruct [25] methodology and FLAN (Fine-Tuned Language Net)–style prompting [37,38], where GPT-4 [39] was guided by 3 randomly selected seed tasks to produce diverse triplets. For long-form or informal content, such as recipes or patient narratives, we applied Dog-Instruct [40] techniques, which use back-translation and structural rewriting to transform text into high-quality instruction-response pairs. For datasets such as Nutribench, customized templates were applied to standardize nutritional tasks.

To ensure topical focus, we applied a diabetes-specific keyword filtering procedure to retain samples relevant to diabetes management. The filtering included terms related to diabetes and glycemic control, such as diabetes, diabetic, glucose, insulin, glycemic, hyperglycemia, hypoglycemia, glycosylated hemoglobin (HbA_1c_), and blood sugar. Diversity was promoted by discarding instructions with a ROUGE-L (Recall-Oriented Understudy for Gisting Evaluation–Longest Common Subsequence) similarity score greater than 0.7 relative to existing samples. Invalid generations were identified and removed based on heuristics, including instructions that were too short (<3 words) or too long (>150 words), outputs that repeated inputs, or tasks requiring unsupported modalities, such as images or graphs. The resulting dataset encompassed a wide range of diabetes-related tasks, including disease definition and explanation, diabetes-friendly dietary recommendations, glycemic trend analysis, and diet optimization.

### Dataset Optimization Strategy

#### Overview

After constructing the diabetes-specific dataset, we implemented a multistage optimization strategy to improve both the quality and use of the instruction-response pairs. This strategy was inspired by recent advances in reflection-tuning [41] and selective reflection-tuning [10], which show that low-quality or poorly aligned samples can undermine instruction tuning by inducing superficial reasoning, ambiguity, or inconsistent model behavior. By adapting these approaches to the diabetes domain, we ensured that our dataset provided clear, challenging, and semantically aligned samples for instruction tuning.

#### Instruction Optimization via IFD

Instruction optimization focused on refining the clarity and difficulty of prompts so that they provided meaningful guidance to the model. We adopted a reflection-based replacement mechanism following [41], in which a teacher model (eg, GPT-4) critically evaluated each instruction and generated improved versions when necessary.

To quantify instructional difficulty from the student’s perspective, we used IFD, a metric that measures how much the presence of an instruction reduces uncertainty in generating the correct response. Intuitively, IFD captures how cognitively demanding it is for the model to follow a given instruction.

Formally, let* f*_*θ*_ denote the student model with parameters *θ*, *x* the instruction, and *y* the corresponding response. The IFD score is defined as:


(1) $${IFD}_{\theta }(y|x)=\frac{ppl(y|x)}{ppl(y)}-\exp\left(L_{\theta }(y|x)-L_{\theta }(y)\right)$$

where *L*_*θ*_(*y*|*x*) denotes the negative log-likelihood loss of generating response *y* conditioned on instruction *x*, and ppl(·) denotes perplexity. A higher IFD score indicates that the instruction substantially influences the generation of the response, suggesting greater instructional difficulty.

For each original instruction-response pair and its reflected counterpart, the student model retained the version with the higher IFD score:


(2) $$\left(x_{1},y_{1})=\arg\max_{\left(x,y\right)}{IFD}_{\theta }(y|x\right)$$

This selection process filtered out overly trivial or redundant instructions, resulting in a dataset that emphasized cognitively meaningful tasks capable of promoting deeper reasoning and robust instruction-following behavior.

#### Response Optimization via r-IFD

While IFD assesses the difficulty of instructions, it does not directly evaluate the clarity or semantic grounding of responses. To address this limitation, we further optimized responses using r-IFD, which measures how easily the original instruction can be inferred from the response alone.

The motivation behind r-IFD is that a high-quality response should clearly and unambiguously reflect the intent of the instruction. Responses that are vague, underspecified, or weakly grounded provide poor learning signals and may hinder generalization.

Formally, r-IFD is defined as:


(3) $${r\text{-}IFD}_{\theta }(x|y)=\frac{ppl(x|y^{\prime })}{ppl(x)}=\exp\left(L_{\theta }(x|y^{\prime })-L_{\theta }(x)\right)$$

where $y^{\prime }$denotes a transformed version of the response used to prompt the model to recover the instruction. A lower r-IFD score indicates that the model can more easily infer the instruction from the response, reflecting stronger semantic alignment and instructional clarity.

For each instruction-response pair after instruction optimization, we selected the response with the lowest r-IFD score:


(4) $$\left(x_{2},y_{2})=\arg\min_{\left(x_{1},y_{1}\right)}{\text{r-IFD}}_{\theta }(x_{1}|y_{1}\right)$$

Based on IFD and r-IFD, we filter the dataset to retain instruction-response pairs characterized by high IFD scores and low r-IFD scores, respectively. Samples falling outside these optimal thresholds are flagged as suboptimal. The prompt instructs the model to revise instructions by improving topic complexity, required level of detail, domain knowledge requirements, ambiguity, and logical reasoning or problem-solving components. Corresponding responses are refined to improve helpfulness, relevance, accuracy, and level of detail while maintaining consistency with the intended instructional objective.

After refinement, IFD and r-IFD scores were recalculated to determine whether the revised instruction-response pairs satisfied the predefined filtering thresholds. Samples that continued to fall outside the acceptable range after refinement were excluded from the final instruction tuning dataset. The selection logic is formalized as follows:


(5) $$\left(x^{\prime },y^{\prime }\right)=\left\{ \begin{array}{ll} \mathrm{Replace}(x,y), & \text{if }{IFD}_{\theta }(y|x)\;\;x\leq 0.45\;and\;{r\text{-}IFD}_{\theta }(x|y)\geq 0.98\\ (x,y), & \text{otherwise} \end{array}\right.$$

**Figure 2.**
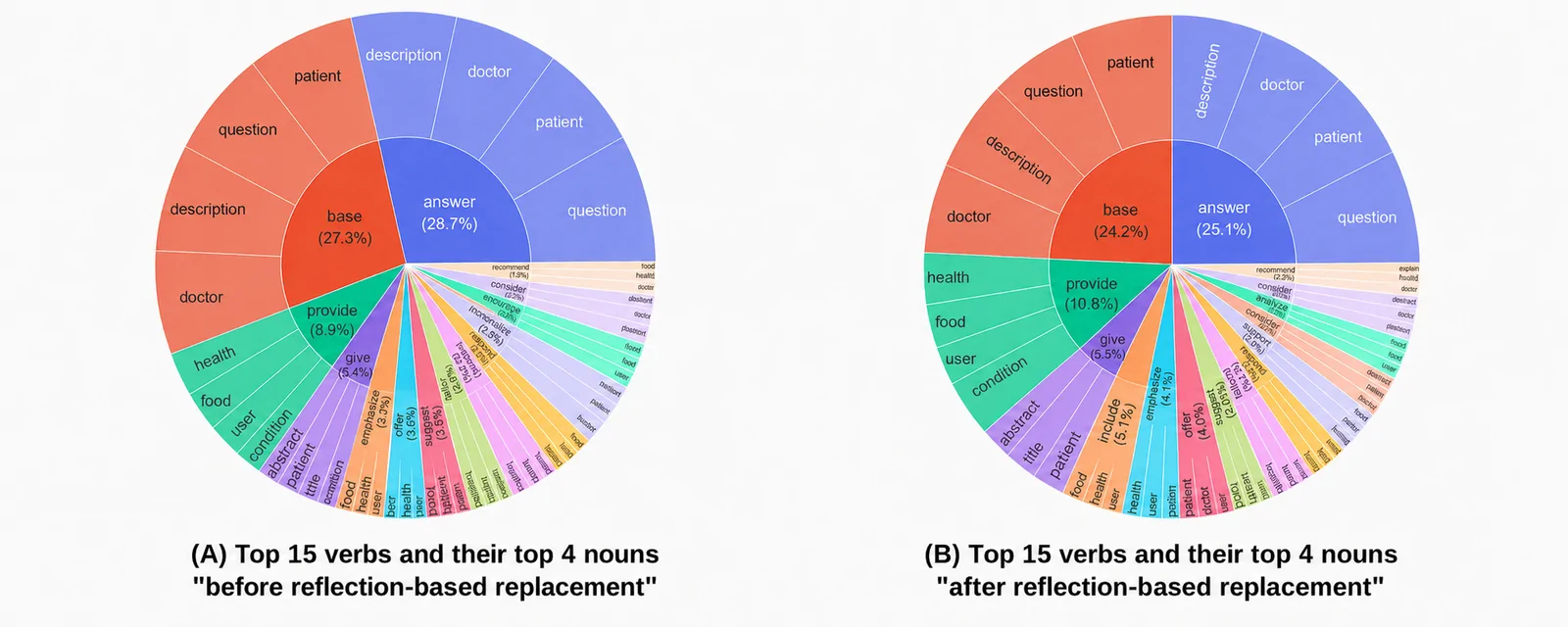
Instruction characteristics before (A) and after (B) reflection-based replacement using instruction-following difficulty and reversed instruction-following difficulty.

Instruction characteristics before and after reflection-based replacement are illustrated in Figure 2, showing a shift toward more diverse and reasoning-oriented instructions following optimization with IFD and r-IFD.

#### Reflection-Based Evaluation Criteria

**Figure 3.**
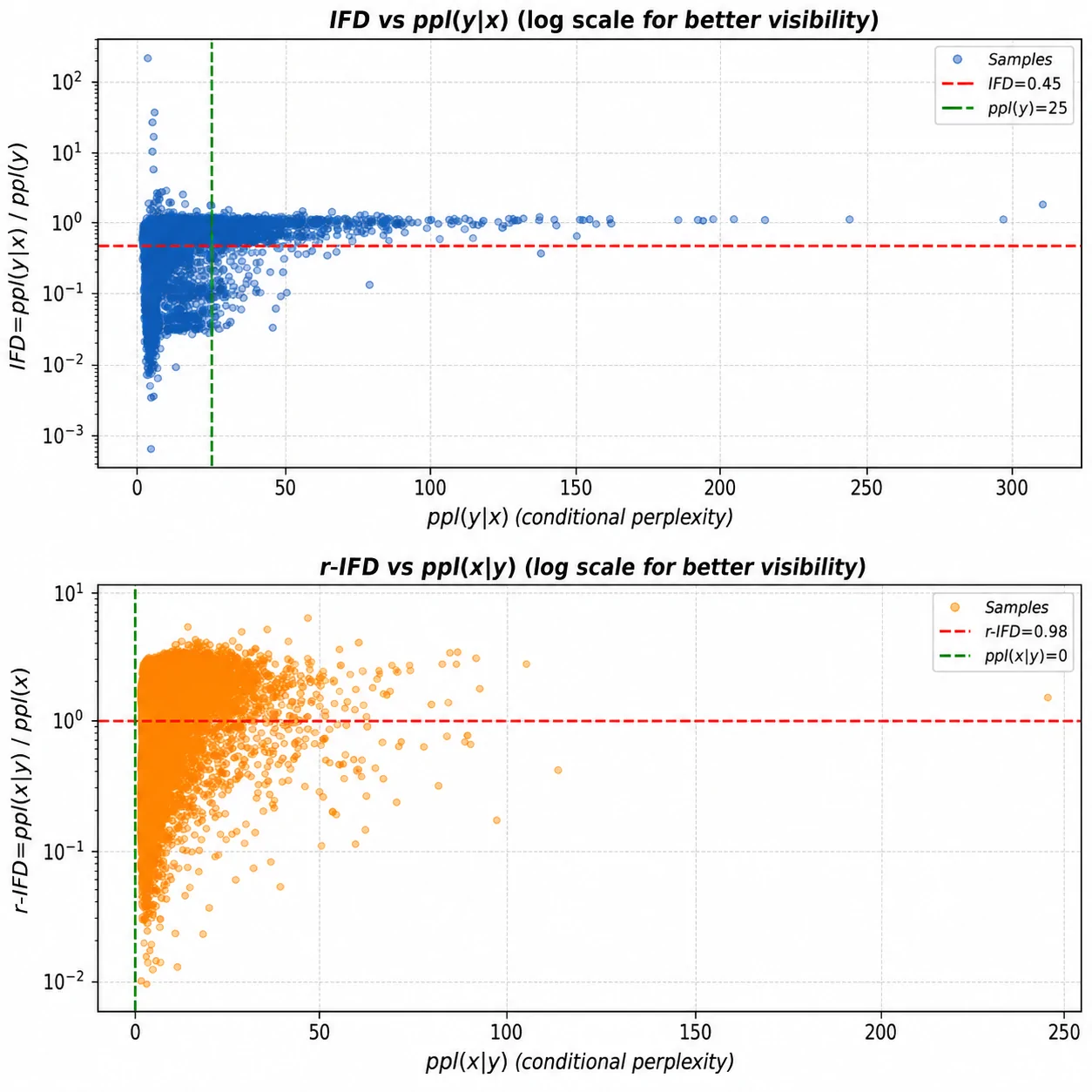
Reflection-based evaluation criteria for instruction dataset optimization. IFD: instruction-following difficulty; r-IFD: revised instruction-following difficulty; ppl: perplexity.

Figure 3 shows the distribution of IFD and r-IFD scores in the instruction dataset. Based on the inspection of the log-scale distributions, we empirically selected threshold values to retain instruction-response pairs consistent with the selected filtering criteria while filtering out ambiguous or weakly instructive samples. This dual-filtering strategy reduced noise in the instruction dataset while preserving task diversity prior to curriculum-based instruction tuning.

### Curriculum-Based Instruction Tuning

Given the heterogeneity of tasks and response lengths, including QA, IE, NLI, summarization, generation, and diabetes-friendly dietary recommendation—the resulting instruction-response pairs vary widely in both structure and response length. Fine-tuning on such heterogeneous data without any ordering can destabilize training and hinder the model’s ability to internalize complex tasks.

**Figure 4.**
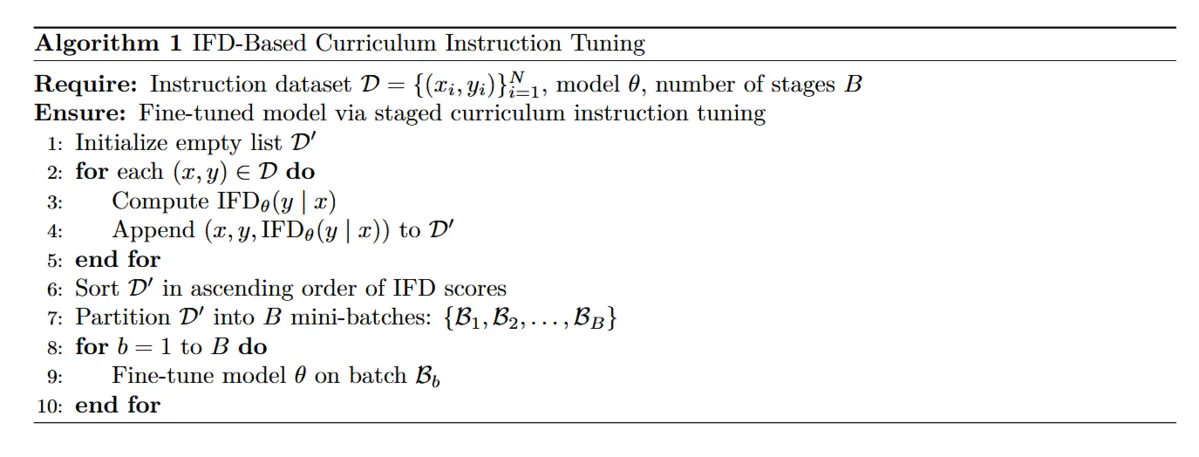
Overview of the reflection-based instruction replacement and curriculum-based instruction tuning procedure used to optimize the diabetes-specific instruction dataset prior to fine-tuning. IFD: instruction-following difficulty.

To address this, we apply a staged curriculum-based instruction tuning strategy that sequences instructions from easier to more challenging, helping the model gradually adapt to increasing task complexity and response diversity. We first rank all instruction-response pairs based on their IFD scores, which reflect the relative difficulty of each sample from the perspective of the student model. An overview of the curriculum-based instruction tuning procedure is shown in Figure 4.

The dataset was subsequently partitioned into minibatches arranged in increasing order of difficulty, where difficulty was quantified using the IFD score. A higher IFD score indicated that the sample was more difficult for the student model to follow. All instruction-response pairs were first sorted in ascending order of the IFD score, from easier to more challenging samples, and then divided into minibatches with similar difficulty levels to minimize intrabatch variance.

Training proceeded sequentially across these ordered minibatches. The model progressed from easier to more difficult samples according to the predetermined IFD-based ordering. This progressive training approach stabilized learning by mitigating task-level heterogeneity and reducing divergence in difficulty across batches.

This facilitated robust convergence and prevented catastrophic forgetting by reinforcing previously acquired general medical competencies before introducing more specialized domain-specific instructions. As a result, the model can effectively acquire expert-level reasoning for diabetes-related tasks without compromising its foundational biomedical knowledge.

### Experimental Setup

In this section, we detail our experimental setup, the benchmark datasets, the baseline models, and the evaluation strategy used to assess the performance and effectiveness of our diabetes-specialized instruction-tuned LLM across multiple diabetes-relevant biomedical NLP tasks and dietary recommendation settings.

### Implementation Details

We instruction-tuned our model using the LLaMA3 architecture [42], experimenting with both 3B- and 8B-parameter variants, and implementing using the *Transformers* library from Hugging Face [43]. To ensure computational efficiency, we used low-rank adaptation [44] via the parameter-efficient fine-tuning framework [45], setting the low-rank adaptation rank to 16, the scaling factor *α* to 16, and the dropout rate to 0.05.

Training was conducted with an effective batch size of 64, achieved using a per-device batch size of 16 and gradient accumulation over 4 steps. We optimized the model using the AdamW optimizer with a learning rate of 2×10^–5^ and trained for 2 epochs. The maximum sequence length was set to 2048 tokens.

All experiments were performed on a single NVIDIA A100 GPU with 40 GB of memory. Instruction filtering and reflection-based replacement were applied before tokenization to ensure alignment between the refined instruction set and the final training batches, including the ordering required for curriculum-based instruction tuning. This preprocessing step ensured consistent batching and preserved the intended difficulty progression during training.

### Benchmark Tasks and Datasets

To align the evaluation benchmarks with the targeted diabetes domain, we constructed diabetes-specific subsets rather than directly using the original open-source benchmark datasets in full. For MedQA-USMLE, MedMCQA, PubMedQA, MedNLI, and the ADE Corpus, candidate samples were first identified using diabetes-related keyword filtering, including terms such as diabetes, diabetic, glucose, insulin, glycemic, hyperglycemia, hypoglycemia, HbA_1c_, and blood sugar. The filtered samples were then manually reviewed to confirm their relevance to diabetes-related clinical knowledge, reasoning, or adverse event extraction. Samples in which diabetes was mentioned only incidentally or that were not directly relevant to diabetes management were excluded from the final evaluation subsets.

The resulting evaluation framework encompasses the following benchmark tasks and datasets:

- Multiple-choice question answering (MCQA) includes the following:
  - MedQA-USMLE [46]: this dataset contains questions based on the US Medical Licensing Examination and is used to evaluate professional-level medical knowledge.
  - MedMCQA [47]: this large-scale dataset contains medical entrance examination questions drawn from various textbooks, with a particular focus on clinical scenarios.
  - PubMedQA [48]: this benchmark evaluates a model’s ability to reason over biomedical abstracts and provide “yes,” “no,” or “maybe” answers.
- NLI includes the following:
  - MedNLI [49]: this dataset uses clinical narratives from MIMIC-III to evaluate a model’s ability to perform medical natural language inference.
- Clinical information extraction (IE) includes the following:
  - ADE Corpus [50]: this corpus is used to evaluate the extraction of adverse drug events and related entities from medical text.
- Medical summarization includes the following:
  - NoteChat [51]: this dataset focuses on summarizing patient-doctor dialogues into structured, professional clinical notes.
- Clinical answer generation includes the following:
  - iCliniq [17]: this real-world doctor-patient question-and-answer corpus is used to assess a model’s ability to provide accurate and compassionate clinical advice.
- Diabetes-specific specialized task includes the following:
  - Nutritional plan generation: this novel task evaluates a model’s ability to generate nutritionally balanced and diabetes-friendly meal plans based on patient-specific constraints.

### Evaluation Metrics

For the classification-based tasks—QA, NLI, and IE—we report accuracy as the primary evaluation metric. Accuracy provides a direct and interpretable measure of task performance across these diabetes-related benchmarks and is commonly used in prior biomedical language model evaluations [34].

For generative tasks, including medical dialog summarization and clinical answer generation, we used a combination of automated semantic similarity metrics and model-based qualitative evaluation. Specifically, we computed BERTScore (Bidirectional Encoder Representations from Transformers Score) [52], which measures semantic similarity between generated outputs and reference texts using contextualized embeddings, and BLEURT (Bilingual Evaluation Understudy with Representations from Transformers) [53], a learned evaluation metric designed to assess both syntactic and semantic alignment with human-written references. These metrics jointly capture content fidelity and linguistic adequacy in long-form medical text generation.

In addition to automated metrics, we evaluated generation quality using GPT-4 as an external evaluator. Each generated output was assessed using 3 criteria: coherence, completeness, and naturalness. Coherence was defined as the logical consistency, contextual relevance, and intersentence connectivity of the response. Completeness was defined as the extent to which the response addressed all clinically relevant aspects of the question without omitting key diabetes-related information. Naturalness was defined as the fluency, readability, and clinical appropriateness of the response, including whether the answer maintained an appropriate balance between patient-friendly language and medical professionalism.

For each evaluation instance, GPT-4 was provided with the input question, the generated response, and the reference answer, where applicable. The prompt included explicit definitions of coherence, completeness, and naturalness, together with a predefined scoring rubric, and GPT-4 was instructed to assign separate scores from 1 to 5 for each criterion. The full GPT-4 evaluation prompts and scoring rubrics used for answer correctness and generation-quality assessment are provided in Multimedia Appendix 1 to improve transparency and reproducibility.

The use of GPT-4 for generation evaluation was motivated by prior studies demonstrating strong alignment between GPT-4–based assessments and human judgments in natural language generation tasks, including clinical dialog and medical text generation. However, no separate human evaluation or human-GPT-4 calibration was conducted in this study.

All evaluations were conducted on held-out test data that were not used during training.

### Baseline Models

To benchmark the performance of our model, we conducted a comparative analysis against several prominent biomedical LLMs, including the following:

- MedAlpaca 7B is an instruction-tuned biomedical language model derived from LLaMA and fine-tuned on medical QA and dialog-style datasets.
- PMC-LLaMA is a domain-adapted LLaMA model pretrained on a large-scale corpus of biomedical literature, including PubMed Central articles and medical textbooks.
- ChatDoctor is a conversational medical language model fine-tuned on synthetic doctor-patient interactions.
- AlpaCare LLaMA2 7B [54] is an instruction-tuned medical assistant model trained on curated health care dialog data.
- BioMistral is a biomedical LLM based on the Mistral-7B architecture, pretrained on PubMed Open Access content.
- ME-LLaMA [55] is a model focused on evidence-based medicine and designed to prioritize grounding in verifiable clinical evidence.

## Results

### Overview

We evaluated the proposed diabetes-specialized instruction-tuned LLM across multiple diabetes-related task categories, including QA, NLI, IE, summarization, generation, and diabetes-friendly dietary recommendation. Performance was compared with several state-of-the-art biomedical and medical instruction–tuned baselines. Unless otherwise stated, all results are reported on held-out test sets not used during training.

### Performance on QA and NLI Tasks

We first evaluated our model on MCQA (MedQA-USMLE, MedMCQA, and PubMedQA) and NLI (MedNLI). Results are summarized in Table 1.

Across QA tasks, the proposed model demonstrated strong performance relative to existing biomedical baselines. It achieved accuracy scores of 0.44 on MedQA-USMLE, 0.53 on MedMCQA, and 0.78 on PubMedQA, yielding the highest average QA performance among all evaluated models. The improvement was particularly pronounced on PubMedQA, where the proposed model exceeded the next-best baseline by a substantial margin, highlighting its enhanced ability to retrieve and reason over diabetes-related biomedical facts.

**Table 1.** Zero-shot and instruction-tuned performance of diabetes-specialized large language models compared to baseline models on MCQA^a^ and NLI^b^ tasks.

Model	MedQA-USMLE^c^	MedMCQA	PubMedQA	MedNLI	ADE^d^ Corpus
MedAlpaca 7B, accuracy	0.34	0.28	0.05	0.55	0.31
PMC^e^-LLaMA, accuracy	0.20	0.14	0.40	0.13	0.02
ChatDoctor, accuracy	0.18	0.17	0.37	0.21	0.21
AlpacaCare LLaMA2 7B, accuracy	0.09	0.35	0.60	0.13	0.22
BioMistral, accuracy	0.36	0.42	0.78	0.48	0.35
Me-LLaMA, accuracy	0.36	0.33	0.26	0.23	0.31
LLaMA3.2 3B (zero-shot), accuracy	0.24	0.30	0.20	0.07	0.29
LLaMA3.2 3B (instruction-tuned), accuracy	0.29	0.31	0.78	0.25	0.34
LLaMA3.1 8B (zero-shot), accuracy	0.34	0.30	0.55	0.21	0.30
LLaMA3.1 8B (ours), accuracy	0.44	0.53	0.78	0.39	0.39

a MCQA: multiple-choice question answering.

b NLI: natural language inference.

c USMLE: US Medical Licensing Examination.

d ADE: adverse drug event.

e PMC: PubMed Central.

For NLI, when evaluated on the MedNLI dataset, the proposed model achieved an accuracy of 0.39. While this performance was slightly lower than that of MedAlpaca 7B, which attained an accuracy of 0.55, this difference aligns with the composition of the training data. Specifically, the diabetes-specific instruction dataset contained a limited number of entailment-focused examples, many of which were filtered during reflection-based replacement due to low instructional value. Despite this, the model maintained competitive NLI performance through broader exposure to reasoning-oriented instructions.

### Performance on IE Tasks

We assessed IE performance using the ADE Corpus, focusing on accuracy as the primary metric. As shown in Table 1, the proposed model achieved an accuracy of 0.39, matching the best-performing baseline, BioMistral.

These results indicate that the diabetes-specialized instruction tuning process preserved the model’s ability to extract structured biomedical information while prioritizing diabetes-specific reasoning and instruction-following. Importantly, the reflection-based replacement strategy did not degrade extraction performance despite selectively refining the instruction dataset.

We carried out additional experiments to evaluate GPT-4o-mini on the same diabetes-specific benchmark subsets. As shown in Table 2, GPT-4o-mini achieved higher performance on QA and NLI tasks, whereas the proposed model achieved substantially higher performance on the ADE IE task.

**Table 2.** Comparison of GPT-4o-mini and the proposed model on diabetes-specific benchmark subsets.

Task	Proposed model (8B), accuracy	GPT-4o-mini, accuracy
MedQA^a^-USMLE^b^	0.44	0.75
MedMCQA^c^	0.53	0.75
PubMedQA	0.78	1.00
MedNLI^d^	0.39	0.75
ADE^e^ (IE^f^)	0.39	0.05

a QA: question answering.

b USMLE: US Medical Licensing Examination.

c MCQA: multiple-choice question answering.

d NLI: natural language inference.

e ADE: adverse drug event.

f IE: information extraction.

### Performance on Medical Dialog Summarization and Answer Generation

Furthermore, we evaluated generative performance on medical dialog summarization (NoteChat) and medical answer generation (iCliniq), and the results are reported in Tables 3 and 4, respectively.

**Table 3.** Performance on medical dialog summarization using the NoteChat dataset.

Model	Coherence	Completeness	Naturalness	BLEURT^a^	BERTScore^b^	Average (SD)
MedAlpaca 7B	0.772	0.470	0.810	0.72	0.61	0.676 (0.123)
PMC^c^-LLaMA	0.013	0	0.130	0.55	0.50	0.239 (0.239)
ChatDoctor	0.405	0.315	0.485	0.41	0.43	0.409 (0.055)
AlpacaCare LLaMA2 7B	0.802	0.675	0.847	0.67	0.62	0.723 (0.086)
BioMistral	0.693	0.682	0.820	0.52	0.73	0.689 (0.097)
Me-LLaMA	0.755	0.645	0.835	0.55	0.64	0.685 (0.099)
LLaMA3.2 3B (zero-shot)	0.320	0.235	0.335	0.41	0.34	0.328 (0.056)
LLaMA3.2 3B (instruction-tuned)	0.817	0.667	0.863	0.61	0.54	0.699 (0.123)
LLaMA3.1 8B (zero-shot)	0.355	0.287	0.382	0.35	0.33	0.341 (0.032)
LLaMA3.1 8B (ours)	0.823	0.693	0.863	0.67	0.65	0.740 (0.086)

a BLEURT: Bilingual Evaluation Understudy with Representations from Transformers.

b BERTScore: Bidirectional Encoder Representations from Transformers score.

c PMC: PubMed Central.

**Table 4.** Performance on medical answer generation using the iCliniq dataset.

Model	Coherence	Completeness	Naturalness	BLEURT^a^	BERTScore^b^	Average (SD)
MedAlpaca 7B	0.63	0.45	0.70	0.56	0.61	0.590 (0.083)
PMC^c^-LLaMA	0.245	0.162	0.38	0.60	0.62	0.401 (0.184)
ChatDoctor	0.62	0.453	0.698	0.57	0.62	0.592 (0.081)
AlpacaCare LLaMA2 7B	0.827	0.665	0.935	0.56	0.44	0.685 (0.178)
BioMistral	0.502	0.312	0.613	0.55	0.52	0.499 (0.101)
Me-LLaMA	0.68	0.483	0.798	0.60	0.67	0.646 (0.103)
LLaMA3.2 3B (zero-shot)	0.745	0.573	0.90	0.56	0.47	0.650 (0.154)
LLaMA3.2 3B (instruction-tuned)	0.775	0.603	0.907	0.69	0.50	0.695 (0.140)
LLaMA3.1 8B (zero-shot)	0.588	0.44	0.645	0.56	0.55	0.557 (0.067)
LLaMA3.1 8B (ours)	0.83	0.655	0.925	0.57	0.63	0.722 (0.133)

a BLEURT: bilingual evaluation understudy with representations from transformers.

b BERTScore: Bidirectional Encoder Representations from Transformers Score.

c PMC: PubMed Central.

On the NoteChat summarization task, the proposed model achieved the highest average score (0.74), outperforming all baseline models across coherence, completeness, and naturalness. Similarly, on the iCliniq clinical answer generation task, the model achieved an average score of 0.722, demonstrating a strong capability in producing clinically appropriate, context-aware, and user-aligned responses.

Automatic semantic similarity metrics (BLEURT and BERTScore) further supported these findings, indicating that the generated outputs closely aligned with reference responses both syntactically and semantically. GPT-4–based evaluations showed that the proposed model maintained fluent and natural language generation while delivering responses that were complete and tailored to diabetes-related clinical contexts.

### Evaluation of Diabetes-Friendly Dietary Recommendations

To assess domain specialization beyond standard NLP tasks, we evaluated the model’s ability to generate diabetes-friendly dietary recommendations. In this setting, we compared the proposed model with GPT-4, a strong general-purpose baseline.

Each model generated 10 full-day meal plans for individuals with diabetes. The generated plans were evaluated using two complementary criteria: (1) language quality, assessed via GPT-4–based coherence, completeness, and naturalness scores; and (2) nutritional quality, assessed using the DQI-I score.

**Figure 5.**
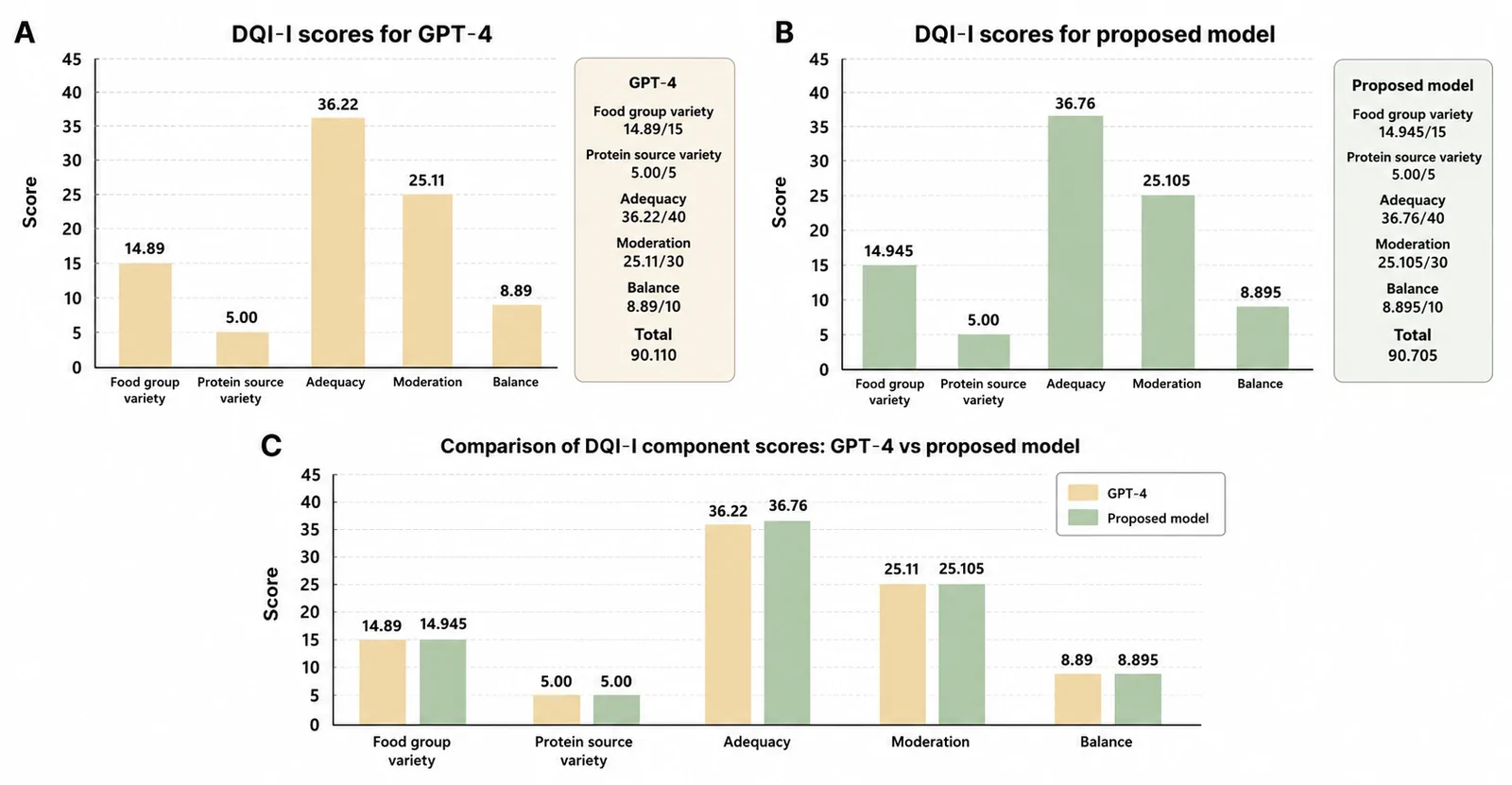
Comparison of Diet Quality Index–International (DQI-I) component scores for diabetes-friendly meal plans generated by GPT-4 and the proposed diabetes-specialized model: (A) DQI-I scores for GPT-4, (B) DQI-I scores for the proposed model, and (C) comparison of DQI-I component scores between GPT-4 and the proposed model.

As shown in Figure 5, the proposed model achieved higher average DQI-I scores than those of GPT-4 across multiple dietary components, indicating superior nutritional balance and closer alignment with dietary guidelines for diabetes management.

Language quality scores are summarized in Table 5.

**Table 5.** Language quality scores using GPT-4–based ratings of coherence, completeness, and naturalness.

Model	Coherence	Completeness	Naturalness
GPT-4	0.847	0.930	0.972
Proposed model	0.825	0.915	0.915

GPT-4 achieved slightly higher scores for coherence, completeness, and naturalness; however, the differences were small, and the proposed model maintained competitive language quality while producing more nutritionally appropriate meal plans for diabetes-specific needs.

Although the improvement in DQI-I was modest, the proposed model generated meal plans with higher nutritional quality and lower simulated postprandial glycemic burden than those generated by GPT-4. This finding suggests that domain-specific instruction tuning may provide benefits for diabetes-focused dietary recommendations despite relatively small differences in language-quality metrics.

### Simulation-Based Evaluation of Glycemic Response

To further validate the physiological relevance of the generated dietary recommendations, we conducted simulation-based evaluations using the SimGlucose simulator, an open-source simulator based on the US Food and Drug Administration–validated UVA/Padova type 1 diabetes model.

We simulated postprandial glycemic responses under 2 conditions: carbohydrate-only intake and carbohydrate intake combined with insulin dosing. In both scenarios, meal plans generated by the proposed model resulted in lower incremental area under the curve values compared with those generated by GPT-4. Specifically, the proposed model achieved a lower postmeal incremental area under the curve (161.53 mg/dL/min) than GPT-4 (163.97 mg/dL/min).

**Figure 6.**
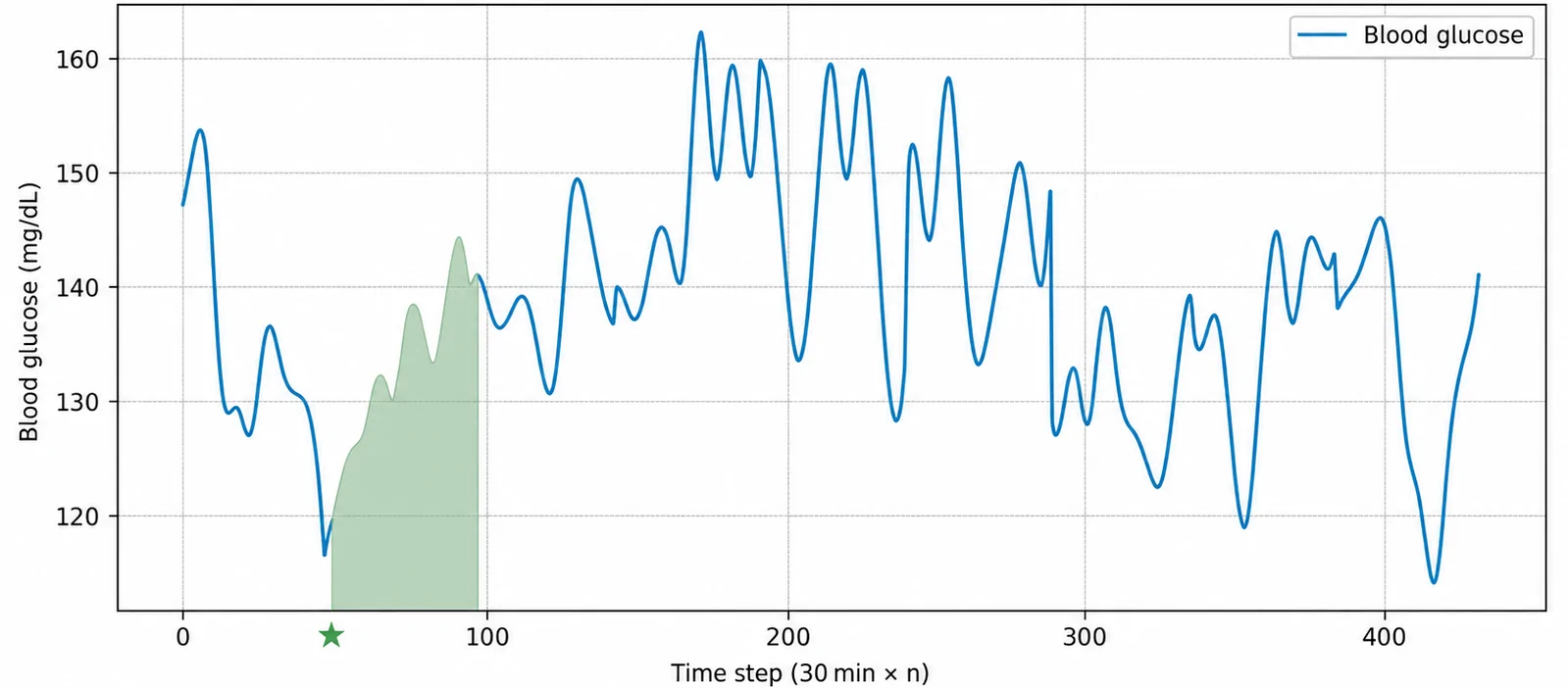
Simulated blood glucose trajectory over a 9-day period without insulin. The shaded star indicates the start of the highlighted interval, and the shaded area represents the incremental area under the curve used to assess the postprandial glycemic response, corresponding to the highlighted window.

The corresponding blood glucose trajectories over a 9-day simulation period without insulin are illustrated in Figure 6, showing reduced glycemic excursions when following meal plans generated by the proposed model. These findings indicate that the model’s dietary recommendations are not only nutritionally aligned but also physiologically favorable for glycemic control. Representative qualitative examples of dietary recommendations, failure cases, carbohydrate distributions, and corresponding glycemic response simulations are provided in Multimedia Appendix 1.

### Ablation Study of Instruction Optimization and Curriculum-Based Instruction Tuning

To directly evaluate the contribution of the proposed instruction optimization and curriculum-based instruction tuning strategies, we conducted an ablation study examining the impact of each major training component. Specifically, we compared the base model with progressively enhanced variants, including instruction tuning with seed diabetes-specific data, IFD-based instruction optimization, combined IFD and r-IFD optimization, and the final curriculum-based instruction tuning strategy. Results are reported across diabetes-related benchmark tasks in Table 6, medical dialog summarization in Table 7, and clinical answer generation in Table 8.

**Table 6.** Ablation study results across diabetes-related benchmark tasks.

Model	MedQA-USMLE^a^	MedMCQA^b^	PubMedQA^c^	MedNLI^d^	ADE^e^ Corpus
LLaMA3.1 8B (zero-shot), accuracy	0.34	0.30	0.55	0.21	0.30
+ Seed, accuracy	0.41	0.37	0.69	0.41	0.32
+ IFD^f^, accuracy	0.47	0.52	0.72	0.40	0.34
+ r-IFD^g^, accuracy	0.43	0.52	0.82	0.37	0.36
+ Curriculum-based instruction tuning, accuracy	0.44	0.53	0.78	0.39	0.39

a USMLE: US Medical Licensing Examination.

b MCQA: multiple-choice question answering.

c QA: question answering.

d NLI: natural language inference.

e ADE: adverse drug event.

f IFD: instruction-following difficulty.

g r-IFD: revised instruction-following difficulty.

**Table 7.** Ablation study results for medical dialog summarization using the NoteChat dataset.

Model	Coherence	Completeness	Naturalness	BLEURT^a^	BERTScore^b^
LLaMA3.1 8B (zero-shot)	0.355	0.287	0.382	0.35	0.33
+ Seed	0.837	0.670	0.853	0.41	0.45
+ IFD^c^	0.780	0.710	0.875	0.52	0.48
+ r-IFD^d^	0.823	0.718	0.875	0.65	0.60
+ Curriculum-based instruction tuning	0.823	0.693	0.863	0.67	0.65

a BLEURT: Bilingual Evaluation Understudy with Representations from Transformers.

b BERTScore: Bidirectional Encoder Representations from Transformers Score.

c IFD: instruction-following difficulty.

d r-IFD: revised instruction-following difficulty.

**Table 8.** Ablation study results for clinical answer generation using the iCliniq dataset.

Model	Coherence	Completeness	Naturalness	BLEURT^a^	BERTScore^b^
LLaMA3.1 8B (zero-shot)	0.588	0.440	0.645	0.56	0.55
+ Seed	0.810	0.608	0.913	0.61	0.67
+ IFD^c^	0.835	0.650	0.927	0.56	0.63
+ r-IFD^d^	0.808	0.635	0.883	0.63	0.57
+ Curriculum-based instruction tuning	0.830	0.655	0.925	0.57	0.63

a BLEURT: Bilingual Evaluation Understudy with Representations from Transformers.

b BERTScore: Bidirectional Encoder Representations from Transformers Score.

c IFD: instruction-following difficulty.

d r-IFD: revised instruction-following difficulty.

Overall, the ablation results show that each component contributed to model performance. IFD-based optimization improved instruction selection by prioritizing samples that were more informative for the student model, while r-IFD-based optimization improved semantic alignment between instructions and responses. Curriculum-based instruction tuning further supported performance by progressively exposing the model to increasingly difficult instruction-response pairs.

## Discussion

### Principal Findings

In this study, we developed and evaluated a diabetes-specialized, instruction-tuned LLM designed to support diabetes-related reasoning, instruction-following, and dietary recommendation tasks. Across a diverse set of diabetes-relevant benchmarks, including QA, NLI, IE, medical dialog summarization, answer generation, and nutrition-focused planning, the proposed model consistently demonstrated strong performance relative to existing biomedical and general-purpose LLMs.

The model achieved strong performance on diabetes-focused QA tasks and generated competitive medical summaries and responses, as measured by both automated metrics and GPT-4–based evaluations of coherence, completeness, and naturalness. In dietary recommendation tasks, the proposed model produced meal plans with higher DQI-I scores than those generated by GPT-4, indicating improved nutritional balance and suitability for diabetes management. Simulation-based evaluation using the SimGlucose simulator further suggested that these meal plans were associated with more favorable postprandial glycemic responses, supporting the physiological plausibility of the generated recommendations.

Importantly, the model maintained competitive performance on general biomedical tasks such as NLI and adverse event IE, suggesting that domain specialization did not substantially degrade broader biomedical capabilities. Together, these results indicate that targeted instruction tuning may improve diabetes-specific performance while preserving broader biomedical capabilities.

### Limitations

This study has several limitations. First, although we evaluated the model across multiple diabetes-related tasks, the benchmarks remain limited to publicly available datasets and simulated scenarios. Real-world clinical deployment would require rigorous prospective evaluation, including clinician oversight and patient safety assessments.

Second, dietary recommendation quality was assessed using established nutritional indices and glycemic simulations rather than by direct clinical outcomes. While these measures provide useful proxies, they cannot fully capture individual variability, comorbidities, or behavioral factors that influence diabetes management.

Third, GPT-4 was used as an evaluator for several generative metrics. Although prior studies have shown strong alignment between GPT-4–based evaluation and human judgment, automated evaluation may still introduce biases or overlook subtle clinical errors. In addition, no separate human evaluation or human-GPT-4 calibration was performed in this study. Future work should include expert clinical evaluation to further validate the reliability of GPT-4–based assessment, particularly for detecting subtle medical inaccuracies or clinically inappropriate recommendations.

Finally, the model was fine-tuned for a specific architecture and parameter scale. The generalizability of the proposed instruction optimization strategy to other model families and larger-scale systems warrants further investigation.

### Comparison With Prior Work

Prior work has demonstrated that general-purpose and biomedical LLMs can support a range of health care–related tasks, including medical QA, dialog summarization, and patient education. However, multiple studies have also reported that these models often struggle with chronic disease management scenarios that require sustained reasoning, contextual interpretation, and domain-specific judgment, particularly in diabetes care.

Unlike earlier approaches that rely primarily on large-scale biomedical pretraining or generic instruction tuning, this study emphasizes model-centric instruction optimization, in which training data are refined based on the student model’s learning behavior. By incorporating reflection-based replacement and curriculum-based instruction tuning, the proposed approach extends prior instruction tuning frameworks by explicitly accounting for instruction difficulty, ambiguity, and learning progression. This contrasts with existing diabetes-related LLM efforts that either focus on narrow tasks (eg, CGM summarization or education chatbots) or depend heavily on retrieval-based augmentation without optimizing instruction-level learning dynamics.

The observed improvements in diabetes-focused reasoning and dietary planning suggest that aligning instruction data with the model’s receptiveness and sequencing tasks by difficulty may provide complementary benefits for domain-specific adaptation.

### Implications for Diabetes Management and Clinical Informatics

The findings of this study have several implications for the development of AI-assisted tools in diabetes care. First, these findings highlight the potential of domain-specialized LLMs to support complex, multifaceted diabetes management tasks that extend beyond factual QA. The ability to generate nutritionally appropriate meal plans, explain glycemic trends, and provide context-aware responses may be particularly valuable for patient education and decision support.

Second, the results suggest that instruction-level data curation and training strategies play a critical role in determining model behavior in clinical domains. Rather than treating instruction datasets as static resources, adaptive refinement based on model feedback may help mitigate common issues, such as vague responses, superficial reasoning, and poor alignment with clinical intent.

Finally, the use of simulation-based evaluation provides a complementary perspective on text-based metrics by assessing downstream physiological plausibility. Although not a substitute for clinical validation, such simulations may help identify potentially harmful or suboptimal recommendations during model development.

### Conclusions

This study presents a diabetes-specialized instruction-tuned LLM optimized through reflection-based replacement and curriculum-based instruction tuning. By aligning instruction data with the model’s learning capacity and sequencing tasks by difficulty, the proposed approach enables robust diabetes-specific reasoning and instruction-following across diverse tasks, including QA, NLI, medical dialog processing, and dietary recommendations.

The model consistently outperformed existing biomedical and general-purpose LLMs on diabetes-focused benchmarks while maintaining competitive performance on general biomedical tasks. Simulation-based evaluation further supported the physiological plausibility of the generated dietary recommendations.

These findings suggest that carefully designed domain-specific instruction tuning can improve the clinical relevance and diabetes-specific capabilities of general-purpose language models for chronic disease management. Future work should focus on prospective clinical evaluation, integration with real-world decision-support systems, and extension of the proposed framework to other chronic conditions.

## Supplementary material

10.2196/92843 Multimedia Appendix 1 Evaluation details, including GPT-4 evaluation prompts and scoring rubrics, and case studies of dietary recommendation and glycemic response simulation.

## Abbreviations

BERTScore: Bidirectional Encoder Representations from Transformers Score
BioGPT: biomedical generative pretrained transformer
BLEURT: Bilingual Evaluation Understudy with Representations from Transformers
CGM: continuous glucose monitoring
DQI-I: Diet Quality Index–International
FLAN: Fine-Tuned Language Net
HbA_1c_: glycosylated hemoglobin
IE: information extraction
IFD: instruction-following difficulty
LLM: large language model
MCQA: multiple-choice question answering
NLI: natural language inference
NLP: natural language processing
QA: question answering
r-IFD: revised instruction-following difficulty
ROUGE-L: Recall-Oriented Understudy for Gisting Evaluation–Longest Common Subsequence
