# Supplementary material for: Reflection- and Curriculum-Based Instruction Tuning for Diabetes-Specialized Large Language Models: Model Development and Evaluation Study
Source: JMIR AI. 2026 Aug 25;5:e92843. doi: 10.2196/92843 (PMC13505602; doi:10.2196/92843)
Supplement: Supplementary file 1 [file ai-v5-e92843-s001.docx]

## Appendix

#### A. Prompt on Win Rate Comparison with GPT-4o versus Our Model.

System prompt:
You are an AI evaluator comparing two model-generated responses to the same prompt. Your task is to determine which response is better overall, considering correctness, relevance, completeness, and clarity.

User prompt:
Compare the following two responses.

Input prompt:
{prompt}

Proposed model response:
{llama3_output}

GPT-4o response:
{gpt4o_output}

Which response is better?

Respond using only one of the following labels:

- Proposed model
- GPT-4o
- Tie

#### B. Prompt on Answer Correctness Evaluation (For QA, NLI tasks – MedQA, MedMCQA, PubMedQA, BioNLI)

System prompt:
You are evaluating the correctness of an AI-generated medical answer.

User prompt:
Compare the correct answer and the model output below. Determine whether the model output conveys the same meaning as the correct answer.

Correct answer:
{true_answer}

Model output:
{model_answer}

If the model output correctly conveys the same answer as the correct answer, respond only with “YES.”
If the model output is incorrect or has a different meaning, respond only with “NO.”

#### C. Prompt on Summarization / Generation Quality Evaluation (For Summarization, Generation, Daily Diets, Alternative Diet)

You are an expert evaluator of medical text generation quality.

User prompt:
Evaluate the model-generated response using the following three criteria:

1. Coherence: The logical consistency, contextual relevance, and inter-sentence connectivity of the response.
2. Completeness: The extent to which the response addresses all clinically relevant aspects of the input without omitting key information.
3. Naturalness: The fluency, readability, and clinical appropriateness of the response, including the balance between patient-friendly language and medical professionalism.

Input:
{input_text}

Model response:
{model_output}

Reference answer:
{true_output}

Please rate each criterion on a scale from 1 to 5, where 1 indicates very poor quality and 5 indicates excellent quality.

Use the following response format only:
Coherence: X
Completeness: X
Naturalness: X

#### Qualitative Case Studies of Dietary Recommendation and Glycemic Response Simulation

To provide additional insight into model behavior, we conducted qualitative analyses of representative dietary recommendations and corresponding glycemic response simulations.

**Case Study 1. Representative Dietary Recommendation**

**Input scenario:**
Adult patient with type 2 diabetes seeking a nutritionally balanced daily meal plan to support glycemic control.

Meal plan generated by the proposed model:

| Meal | Menu |
| --- | --- |
| Breakfast | Ginger-infused oatmeal |
| Lunch | Grilled chicken salad |
| Dinner | Orange-rosemary glazed salmon with broccolini |

This case illustrates a representative meal plan generated by the proposed model.

**Case Study 2. Failure Case**

**Input scenario:**
Adult patient with type 2 diabetes seeking a diabetes-friendly daily meal plan.

Meal plan generated by the proposed model:

| Meal | Menu |
| --- | --- |
| Breakfast | Strawberry Banana “Ice Cream” |
| Lunch | Chicken and Cucumber Lettuce Wraps with Peanut Sauce |
| Dinner | Orange-rosemary glazed salmon with broccolini |

This case illustrates a limitation of the proposed model. Although the meal plan included generally healthy foods, the breakfast item was a fruit-based dessert with relatively high sugar content, which may be suboptimal for glycemic control

**Carbohydrate Distribution Comparison:**

Representative carbohydrate distributions across generated meal plans are summarized below.

| Model | Breakfast(g) | Lunch(g) | Dinner(g) | Daily Average(g) |
| --- | --- | --- | --- | --- |
| Proposed Model | 36~59 | 10~60 | 25~50 | ~113 |
| GPT-4 | 13~61 | 12~68 | 23~35 | ~119 |

The proposed model showed a more uniform dinner carbohydrate range, whereas GPT-4 showed greater lunch variability. In the corresponding simglucose simulation, the proposed model–generated meal plans were associated with lower postprandial iAUC than GPT-4-generated meal plans.
